# Variety Effect on Peelability and Mechanisms of Action of Late-Ripening Citrus Fruits

**DOI:** 10.3390/plants14091349

**Published:** 2025-04-29

**Authors:** Ya Yuan, Ziyi Huang, Yihong Wang, Lijun Deng, Tie Wang, Defa Cao, Ling Liao, Bo Xiong, Meiyan Tu, Zhihui Wang, Jun Wang

**Affiliations:** 1 College of Horticulture, Sichuan Agricultural University, Chengdu 611130, China; yuanya12321@163.com (Y.Y.); huangziyiyx2024@163.com (Z.H.); 13502145088@163.com (Y.W.); denglijun0919@163.com (L.D.); wangtie106@163.com (T.W.); 19108095703@163.com (D.C.); liao19910331@163.com (L.L.); xiongbo1221@sicau.edu.cn (B.X.); 2 Institute of Horticulture, Sichuan Academy of Agricultural Sciences Key Laboratory of Horticultural Crop Biology and Germplasm Creation in Southwest China, Ministry of Agriculture and Rural Affairs, Chengdu 610066, China; tumeiyan@scsaas.cn

**Keywords:** late-ripening citrus, peel properties, peelability

## Abstract

Peelability, a crucial commercial trait for fresh-eating citrus, has received limited research attention regarding its underlying mechanisms. This study investigated three late-maturing citrus cultivars, namely ‘Qingjian’ (QJ), ‘Mingrijian’ (MRJ), and ‘Chunjian’ (CJ), analyzing their peelability development using texture analysis and exploring the physiological and biochemical factors influencing peeling difficulty. The results showed that peelability improved with fruit maturation, reaching its peak at full ripeness, with the following order of peeling difficulty: QJ (hardest) > MRJ (intermediate) > CJ (easiest). At full maturity, QJ (the most difficult to peel) exhibited more regularly shaped peel cells with fewer intercellular spaces, lower intracellular organic matter accumulation, and higher levels of cell wall polysaccharides, calcium (Ca), and abscisic acid (ABA). These characteristics may be linked to the lower relative expression of soluble sugar (TS)-related genes (*CCR4A*, *SPP1*) and the titratable acid (TA)-related gene (*CsCit1*), as well as the higher relative expression of ABA biosynthesis genes (*NCED1*, *NCED2*). Correlation analyses demonstrated that citrus peel firmness and adhesion strength are significantly associated with multiple growth and developmental characteristics, including fruit morphometric parameters, peel cellular architecture, intracellular organic compound content, cell wall polysaccharide levels and related degradative enzyme activities, calcium concentrations, and endogenous phytohormone profiles. These findings provide valuable insights for studying peelability mechanisms and improving fruit quality in citrus breeding.

## 1. Introduction

Citrus (*Citrus reticulata Blanco*), as the world’s most important fresh fruit category, holds significant economic and ecological value [1]. In recent decades, global citrus breeding programs have undergone notable structural changes, with quality improvement increasingly focusing on seedlessness, easy peelability, intense flavor, and rich aroma [2]. Peelability refers to the ease of separating the rind from the segment membranes. Market research in citrus production and sales reveals that, given similar quality traits, consumers strongly prefer easy-peeling cultivars due to convenience and safety considerations [3,4]. Previous studies have established relatively mature subjective evaluation systems for citrus peelability based on empirical assessments and manual peeling difficulty. For instance, Goldenberg [5] classified 46 citrus cultivars into five grades (1, extremely difficult, to 5, extremely easy), while Simons [6] categorized eight citrus cultivars into two levels: low-grade (rind tightly adhering to flesh, difficult to peel) and high-grade (loose connection between rind and flesh, easy to peel). With recent advancements in texture analyzers and other biomechanical instruments, peelability evaluation has progressively shifted toward objective, scientific, and standardized methodologies. Notably, Yu [7] successfully quantified peeling difficulty by decomposing manual peeling into two measurable parameters using texture analyzers and digital force gauges: rind adhesion strength and penetration resistance (rind hardness).

The citrus peel comprises three histologically distinct layers from exterior to interior: the exocarp (flavedo), mesocarp (albedo), and endocarp [8,9,10]. The penetration resistance of citrus peel primarily originates from the oil gland layer (essential oil-containing structures), exhibiting significant correlation with parenchyma textural modifications, whereas peel adhesion strength is predominantly governed by the spongy layer (albedo tissue), whose histoarchitectural characteristics directly mediate rind-segment membrane binding dynamics [11,12]. The cell, as the fundamental structural unit of fruit tissues, directly determines fruit mechanical properties and texture through its morphology, architecture, spatial arrangement, and integrity [13]. Current scientific consensus holds that fruits with uniform polyhedral cells in compact arrangements exhibit firm texture, high hardness, and crispness, whereas those with irregular cell sizes and large intercellular spaces develop soft, coarse textures [14,15].

The accumulation and compositional changes of intracellular organic solutes can reduce fruit osmotic potential, regulate osmotic homeostasis, influence cell membrane stability, and ultimately modify fruit textural properties [16,17]. Notably, starch serves dual physiological roles: beyond providing essential energy for plant growth and development [18], it functions as a crucial organic osmolyte that maintains cellular turgor pressure, provides mechanical support, and retards firmness loss during fruit ripening [19,20]. The maturation process involves starch hydrolysis into soluble sugars, concomitant with dynamic changes in sugar and organic acid accumulation. These metabolic shifts not only determine flavor profiles but also significantly impact cellular osmotic regulation [21]. However, the complex interactions among starch, sugars, and acids as intracellular organic components remain poorly understood. While current research primarily focuses on sugars and acids as flavor determinants in citrus, their combined effects with starch on peelability have not been systematically investigated.

The plant cell wall plays essential roles in maintaining cellular morphology and size, preserving intercellular adhesion and rigidity, and withstanding turgor pressure [22,23,24]. Structurally composed of the middle lamella, primary wall, and secondary wall, its main constituents include pectins, cellulose (CL), and hemicellulose (HCL) [25,26,27]. Current scientific consensus holds that elevated activities of cell wall polysaccharide-degrading enzymes—particularly cellulase (Cx), pectin methylesterase (PME), and pectin lyase (PL)—induce textural modifications in citrus fruits by altering the CL-HCL matrix architecture and degrading pectinaceous compounds in the middle lamella, thereby directly influencing peelability [28,29]. Recent studies have partially elucidated the impact of cell wall polysaccharides on citrus peelability.

Mineral elements and phytohormones critically regulate cellular architecture and fruit textural modifications. Calcium (Ca), as a fundamental structural component of cell walls, primarily exists in the form of calcium pectate within the middle lamella and cell walls. This configuration enhances intercellular adhesion while improving cellular toughness and elasticity, thereby stabilizing tissue structure [30]. Endogenous phytohormones—including ethylene (ETH), abscisic acid (ABA), gibberellins (GA), and auxins (IAA)—play pivotal regulatory roles in citrus fruit textural dynamics and ripening processes. However, the mechanistic contributions of calcium and these phytohormones to peelability development remain underexplored.

The late-maturing citrus cultivars ‘Qingjian’ (QJ), ‘Mingrijian’ (MRJ), and ‘Chunjian’ (CJ) have become predominant commercial cultivars in China due to their distinctive flavor profiles and favorable market timing. Our preliminary investigations revealed significant variations in manual peelability among these closely related cultivars. This study quantitatively characterized peelability development by measuring pericarp hardness and adhesive strength using a texture analyzer, establishing peeling difficulty classifications at maturity. Through comprehensive analyses of fruit growth characteristics, pericarp cell morphology, intracellular organic compounds, cell wall polysaccharides and degrading enzymes, mineral elements, phytohormones, and differential gene expression across pericarp tissues, we systematically elucidated the physiological and molecular mechanisms underlying peelability variation in late-maturing citrus, providing a foundation for further research on peelability formation.

## 2. Results

### 2.1. Comparison of Fruit External Quality

**Figure 1:**
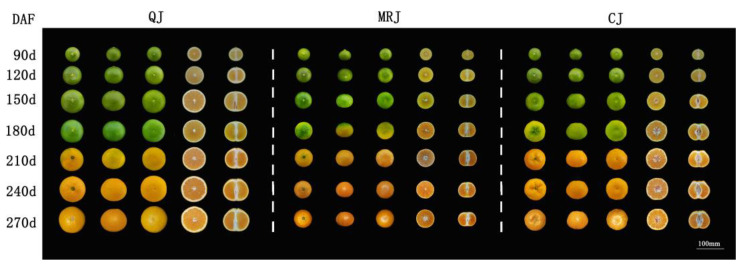
Changes in fruit phenotype of three late-maturing citrus cultivars at different growth and development stages.

Changes in the external fruit quality of three late-maturing citrus cultivars (QJ, MRJ, and CJ) during different growth stages are shown in Figure 1 and Table 1. During 120–180 days after full bloom (DAF), all the cultivars showed rapid fruit expansion, with progressive increases in longitudinal diameter, transverse diameter, and pulp diameter, while maintaining relatively stable peel thickness. Concurrently, the peel color transitioned from dark green to orange–yellow, accompanied by the progressive intensification of pulp coloration. At full maturity (270 d), all the dimensional parameters reached their maximum values, with QJ fruits demonstrating a significantly greater longitudinal diameter, transverse diameter, and pulp diameter compared to both the MRJ and CJ cultivars.

### 2.2. Peelability Comparison of Citrus Fruits

**Figure 2:**
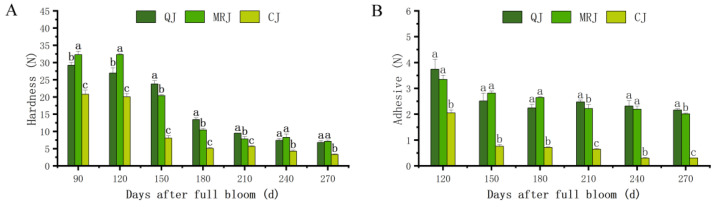
Changes in peel hardness and peel adhesion force of three late-maturing citrus cultivars at different growth and development stages. (**A**) Hardness. (**B**) Adhesive. The parameter values shown in each figure are expressed as mean ± standard deviation (n = 3). Different lowercase letters above the data of different varieties in the same period indicate statistically significant differences as determined by Duncan’s test (*p* < 0.05).

Changes in the peel firmness and peel adhesion of three late-maturing citrus cultivars (QJ, MRJ, and CJ) during different growth stages are shown in Figure 2. A texture analysis demonstrated that both peel firmness and adhesion reached their maximum levels during the early fruit development stage, followed by a progressive decline to their lowest values at full maturity. The most substantial reductions in peel firmness and adhesion occurred during 150–210 and 120–150 d, respectively (peel adhesion measurements commenced at 120 d due to the technical challenge of separating peel from pulp at 90 d). At full maturity, CJ exhibited significantly lower peel firmness compared to both QJ and MRJ, while no significant difference was observed between QJ and MRJ. In terms of peel adhesion, MRJ displayed intermediate values, being significantly lower than QJ but significantly higher than CJ.

### 2.3. Comparison of Peel Characteristics and Cellular Morphology

**Figure 3:**
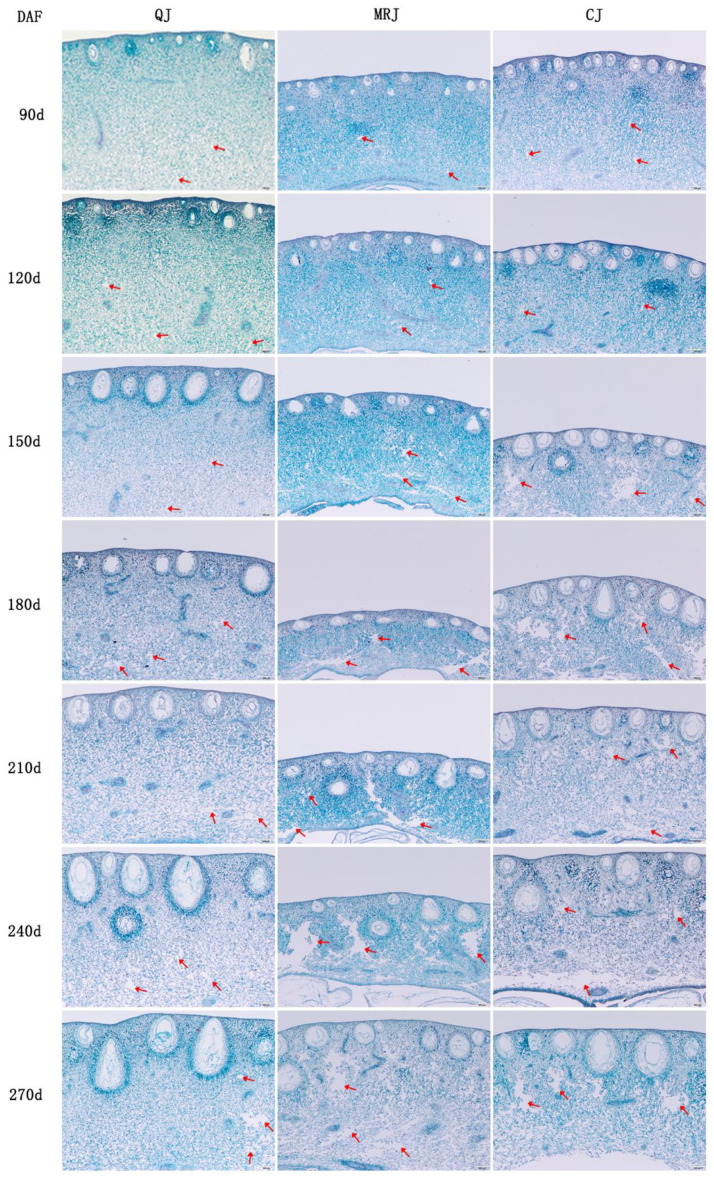
Changes in peel traits of three late-maturing citrus cultivars at different growth and development stages. The area indicated by the red arrow represents the intercellular spaces within the pericarp tissue.

Developmental changes in the peel characteristics and cellular morphology of three late-maturing citrus cultivars (QJ, MRJ, and CJ) are illustrated in Figure 3, revealing distinct temporal patterns in the peel cellular architecture. Throughout the growth period, all the cultivars exhibited the progressive enlargement of oil glands, parenchyma cells, and spongy layer cells, though with marked structural differences: QJ maintained regular cell morphology with tightly packed spongy layer organization, showing only minimal intercellular spaces except for limited conspicuous gaps at full maturity, whereas MRJ and CJ displayed progressive cellular disorganization from 150 to 270 d, characterized by irregular cell shapes, increasingly loose arrangements, and expanded, unevenly distributed intercellular spaces that were macroscopically visible as prominent gaps in equatorial cross-sections. At full maturity, quantitative comparisons showed QJ with a significantly larger oil gland perimeter and area (*p* < 0.05) compared to both MRJ and CJ.

### 2.4. Comparison of Intracellular Organic Matter and Component Contents

**Figure 4:**
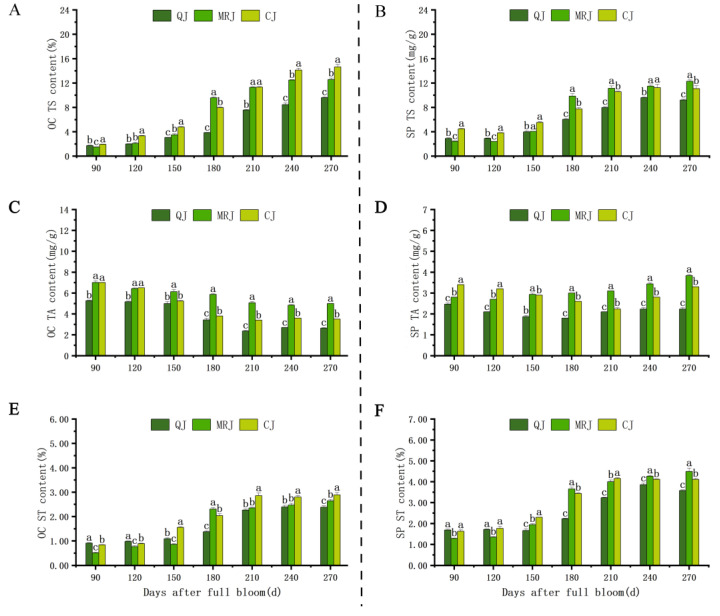
Changes in intracellular organic matter content in the peel of three late-maturing citrus cultivars at different growth and development stages. (**A**) Oil gland layer total sugar content. (**B**) Spongy layer total sugar content. (**C**) Oil gland layer total acid content. (**D**) Spongy layer total acid content. (**E**) Oil gland layer starch content. (**F**) Spongy layer total starch content. Different lowercase letters above the data of different varieties in the same period indicate statistically significant differences as determined by Duncan’s test (*p* < 0.05).

**Figure 5:**
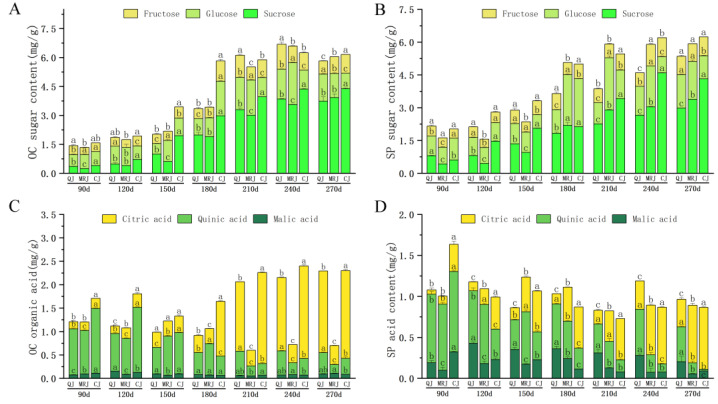
Changes in sugar and acid component content in the peel of three late-maturing citrus cultivars at different growth and development stages. (**A**) Oil gland layer sugar content. (**B**) Spongy layer sugar content. (**C**) Oil gland layer acid content. (**D**) Spongy layer acid content. Different lowercase letters above the data of different varieties in the same period indicate statistically significant differences as determined by Duncan’s test (*p* < 0.05).

The dynamic changes in intracellular organic components within the peel of three late-maturing citrus cultivars (QJ, MRJ, and CJ) during different developmental stages are presented in Figure 4 and Figure 5. Throughout fruit development, all the cultivars exhibited similar trends in component accumulation: both the oil gland layer and spongy layer showed an initial increase followed by stabilization in their total soluble solids (TS) and soluble tannins (ST) contents. The titratable acidity (TA) content in the oil gland layer initially decreased and then increased, while in the spongy layer it exhibited an overall gradual decline throughout the developmental stages. The critical phase of 150–210 d represented a pivotal metabolic transition period, during which the rapid accumulation of TS and ST occurred concurrently with a sharp reduction in the TA content, specifically within the oil gland layer. At full maturity, sucrose and citric acid emerged as the dominant components of TS and TA pools in both tissue layers across all the cultivars. A comparative analysis revealed that QJ consistently maintained significantly lower (*p* < 0.05) intracellular accumulation levels of the TS, ST, and TA components relative to both the MRJ and CJ cultivars, suggesting fundamental differences in carbon partitioning and organic acid metabolism during fruit ripening.

### 2.5. Comparison of Cell Wall Polysaccharide Content and Related Degrading Enzyme Activities

**Figure 6:**
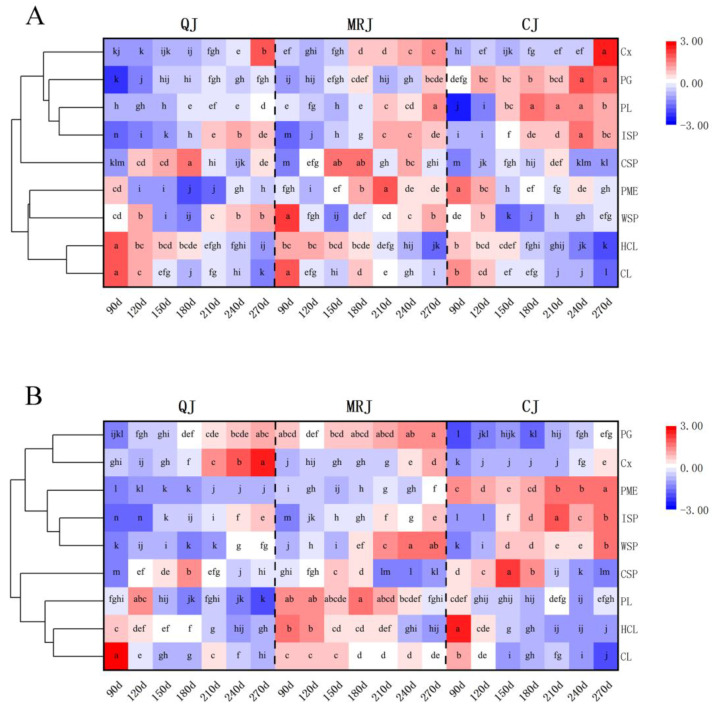
Changes in cell wall polysaccharide content and related degrading enzyme activities in the peel of three late-maturing citrus cultivars at different growth and development stages. (**A**) Oil gland layer. (**B**) Spongy layer. The data means were analyzed and visualized using the Heat Map with Dendrogram tool in Origin software. Different lowercase letters indicate statistically significant differences (*p* < 0.05) according to Duncan’s multiple range test.

Changes in cell wall polysaccharide content and associated degrading enzyme activities in the peel of QJ, MRJ, and CJ during different developmental stages are shown in Figure 6. Throughout fruit development, all three cultivars exhibited consistent trends: The CL and HCL contents gradually decreased in both the oil gland layer and the spongy layer, while cellulase (Cx) and polygalacturonase (PG) activities progressively increased. From 150 to 270 d, the water-soluble pectin (WSP) content and the ion-bound pectin (ISP) content showed continuous accumulation, contrasting with the gradual decline in the chelator-soluble pectin (CSP) content. At full maturity, CJ displayed significantly lower (*p* < 0.05) CL, HCL, and CSP contents in both tissue layers compared to QJ and MRJ, while maintaining higher ISP accumulation. Notably, QJ exhibited significantly reduced (*p* < 0.05) pectate lyase (PL) and pectin methylesterase (PME) activities relative to both MRJ and CJ. Conversely, CJ demonstrated significantly enhanced (*p* < 0.05) Cx and PG activities in the oil gland layer compared to the other cultivars.

### 2.6. Comparison of Ca Content

**Figure 7:**
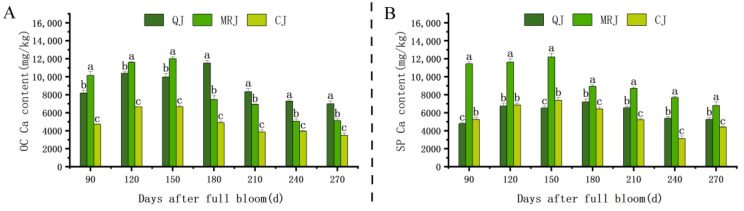
Changes in calcium content in the peel of three late-maturing citrus cultivars during different developmental stages. (**A**) Oil gland layer. (**B**) Spongy layer. Different lowercase letters above the data of different varieties in the same period indicate statistically significant differences as determined by Duncan’s test (*p* < 0.05).

The dynamic changes in Ca content within the peel of QJ, MRJ, and CJ during different developmental stages are presented in Figure 7. All three cultivars exhibited a consistent pattern of initial accumulation followed by gradual reduction in the Ca content across both the oil gland layer and the spongy layer, with the transition point occurring at approximately 180 d. This phase marked the beginning of significant Ca remobilization from peel tissues. At full maturity, the comparative analysis revealed that CJ maintained significantly lower (*p* < 0.05) Ca concentrations in both tissue layers relative to QJ and MRJ. The differential Ca retention capacity among the cultivars may contribute to observed variations in peel structural integrity and postharvest quality, as calcium plays crucial roles in cell wall stabilization and membrane function.

### 2.7. Comparison of Phytohormone Contents in Three Late-Maturing Citrus Cultivars

**Figure 8:**
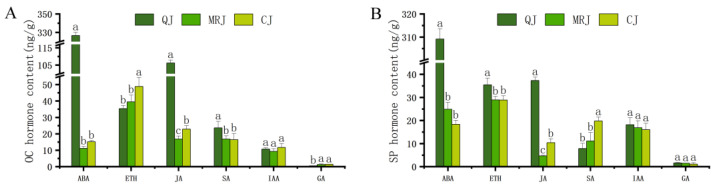
Changes in mesoelement contents in peel tissues of three late-maturing citrus cultivars at full maturity. (**A**) Oil gland layer. (**B**) Spongy layer. Different lowercase letters above the data of different varieties in the same period indicate statistically significant differences as determined by Duncan’s test (*p* < 0.05).

The dynamic changes in phytohormone contents within the peel of QJ, MRJ, and CJ at full maturity are presented in Figure 8. The ABA and ETH concentrations were highest across all the cultivars in both layers, followed by jasmonic acid (JA), salicylic acid (SA), and indole-3-acetic acid (IAA), with GA showing the lowest levels. Specifically, QJ’s oil gland layer contained significantly higher ABA, JA, and SA but lower GA compared to MRJ and CJ (*p* < 0.05). In the spongy layer, QJ exhibited significantly greater ABA, ETH, and JA contents than the other cultivars (*p* < 0.05). However, the IAA levels showed no significant differences in either layer among the three late-maturing citrus cultivars (*p* > 0.05).

### 2.8. Correlation Analysis of Peelability Determinants

**Figure 9:**
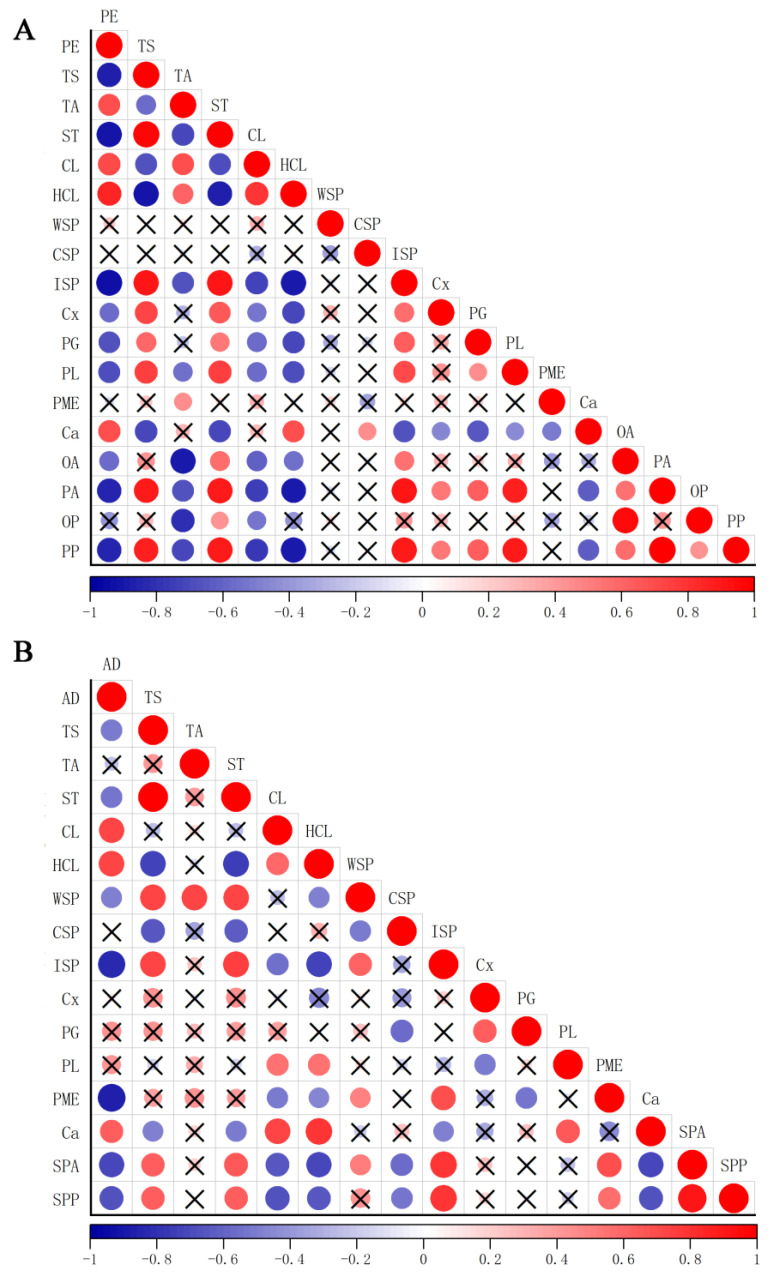
Correlation analysis of peel hardness and peel adhesion force with selected parameters at different growth and development stages. (**A**) Oil gland layer. (**B**) Spongy layer. PE: peel hardness; AD: peel adhesion.

The correlation analysis was conducted on key physiological indicators potentially influencing peelability formation in QJ, MRJ, and CJ cultivars throughout their developmental stages, based on current experimental data and previous studies on citrus peelability and fruit texture, as shown in Figure 9. The results demonstrated that peel hardness showed significant positive correlations with the TA, CL, HCL, and Ca contents in the oil gland layer (*p* < 0.05), while exhibiting significant negative correlations with the TS, ST, and ISP contents and the activities of Cx, PG, and PL (*p* < 0.05). No other indicators showed significant correlations with peel hardness. Similarly, peel adhesion was significantly positively correlated with the CL, HCL, and Ca contents in the spongy layer (*p* < 0.05), but negatively correlated with the TS, ST, WSP, and ISP contents and PME activity (*p* < 0.05), with no significant correlations observed for the other measured parameters.

### 2.9. Comparative Analysis of Gene Expression Patterns in Late-Maturing Citrus Cultivars

**Figure 10:**
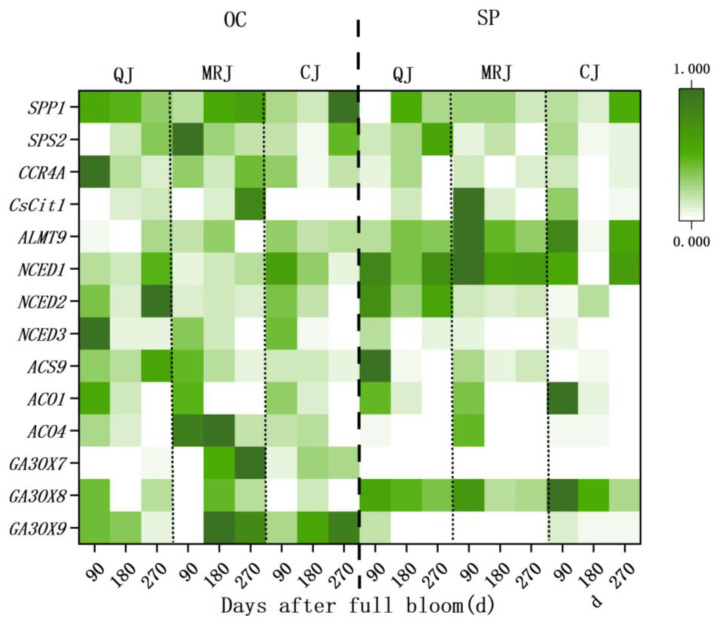
Changes in gene expression levels in the peel of three late-maturing citrus cultivars at different growth and development stages. OC: oil gland layer. SP: spongy layer. The mean values of relative expression data were normalized and analyzed using Origin software.

The gene expression profiles of TS, TA, and phytohormone biosynthesis-related genes were analyzed in the oil gland layers and the spongy layers of QJ, MRJ, and CJ cultivars at 90, 180, and 270 d using quantitative PCR in Figure 10. The results revealed distinct spatiotemporal expression patterns among the cultivars. At full maturity, QJ showed significantly higher expression levels of the TS synthesis gene *ALMT9* and the ABA biosynthesis genes *NCED1* and *NCED2* in the oil gland layers, while exhibiting lower expression of the GA biosynthesis genes *GA30X7* and *GA30X9*. Conversely, in the spongy layers, QJ demonstrated reduced expression of the ABA biosynthesis genes and the TA synthesis gene *CCR4A* compared to the other cultivars. These differential gene expression patterns provide molecular evidence for the observed physiological variations in peel characteristics during fruit maturation.

## 3. Discussion

### 3.1. Influence of Developmental Stages and Cellular Morphology on Peelability Formation in Late-Maturing Citrus Fruits

The maturation of citrus fruits is accompanied by continuous changes in fruit size, color, flavor, and peelability [31]. This experimental study found that the peel hardness and peel adhesion of QJ, MRJ, and CJ gradually decreased with fruit development and ripening, reaching their lowest values at full maturity, which represented the easiest peeling period for these three late-maturing citrus cultivars during the observation phase. These findings are consistent with previous research [32], with varietal differences showing QJ as the most difficult to peel, MRJ as the intermediate, and CJ as the easiest to peel.

The geometric characteristics of fruits, including their cellular arrangement, porosity, and tissue structure, significantly influence textural changes [33,34], as demonstrated by studies showing that apple fruit firmness correlates with parenchyma cell size, shape, intercellular strength, and bonding force in flesh tissue [35]. Our experimental results reveal that the peel cell perimeter and area progressively increase during fruit development and maturation, with QJ exhibiting larger oil glands but smaller, more uniform parenchyma and spongy layer cells with a regular morphology and more compact arrangement at full maturity compared to MRJ and CJ. These findings align with previous research demonstrating that exogenous calcium application promotes tighter cellular arrangement and reduced intercellular spaces in ‘Bing tang’ sweet orange, thereby enhancing fruit firmness and reducing cracking incidence [36]. The results collectively indicate that variations in peel characteristics among late-maturing citrus cultivars, combined with maturation-induced modifications in cellular morphology and arrangement, contribute to reduced peel hardness and adhesion, ultimately developing easy-peeling properties, while the distinct cellular morphology and arrangement patterns at full maturity account for the peelability differences observed among these late-maturing citrus cultivars.

### 3.2. Influence of Intracellular Organic Matter Accumulation on Peelability in Late-Maturing Citrus

The expansion of cell volume requires coordination between cell expansion rates and solute transport/accumulation. Increased solute accumulation and decreased solute potential lead to reduced turgor pressure, enhanced membrane permeability, and cell wall relaxation [37]. Soluble sugars, organic acids, and starch not only contribute to citrus fruit flavor but also serve as key solutes influencing fruit texture, with sucrose, glucose, and fructose being the primary osmotic regulators [38].

Our study revealed that during the observation period, TS and ST accumulation in both the oil gland and spongy layers of QJ, MRJ, and CJ increased as peelability improved, whereas TA accumulation in the spongy layer decreased with reduced peeling difficulty. These findings align with the upregulation of TS synthesis genes (*CsCit1* and *ALMT9*) and the downregulation of TA synthesis genes (*SPP1* and *CCR4A*) in QJ, which exhibited the most challenging peelability. During fruit maturation, the principal components of TS in the oil gland layer and spongy layer of QJ, MRJ, and CJ gradually shifted from glucose to sucrose, while the dominant constituent of TA transitioned from quinic acid to citric acid. At full maturity, sucrose and citric acid were the predominant TS and TA components in both tissue layers across all the cultivars, with QJ showing significantly lower TS, TA, and ST contents compared to MRJ and CJ, consistent with previous findings by Danilo et al. [39,40,41]. The results demonstrate that the accumulation of TS, TA, and ST cellular organic matter, combined with changes in the major components of TS and TA, may collectively reduce the osmotic potential and affect the membrane permeability. These changes decrease the cellular stability in the peels of QJ, MRJ, and CJ citrus varieties, leading to structural alterations that gradually develop easy-peeling characteristics. Moreover, differences in intracellular organic matter accumulation during full ripening cause variations in fruit peelability.

### 3.3. Influence of Cell Wall Polysaccharide Content and Related Degrading Enzyme Activities on Peelability in Late-Maturing Citrus

The plant cell wall is a complex structure primarily composed of cellulose, hemicellulose, and pectin polysaccharides, along with a small number of structural proteins, which collectively provide crucial mechanical support to plant cells [42,43]. Modifications in cell wall architecture and the depolymerization of its structural components are intimately linked to fruit softening, with this process being predominantly driven by the enzymatic degradation of polysaccharides, mediated by various cell wall hydrolases [44]. Throughout fruit development, the observed reduction in peel hardness and adhesion was associated with progressive decreases in CL and HCL content, coupled with elevated activities of cell wall-degrading enzymes, including Cx, PG, PME, and PL: findings that align with the established literature [17]. At full maturity, the comparative analysis revealed that CJ contained significantly lower CL and HCL levels in both the oil gland layer and the spongy layer compared to MRJ and QJ. These results demonstrate that the maturation-associated activation of Cx, PG, and other cell wall-modifying enzymes leads to progressive polysaccharide decomposition, resulting in cell wall loosening that reduces peel hardness and adhesion while enhancing peelability. Furthermore, the cultivar-specific variations in cell wall polysaccharide content and enzymatic degradation profiles ultimately determine the distinct peelability characteristics observed among these late-maturing citrus cultivars at commercial maturity.

### 3.4. Influence of Peel Ca Content on Peelability in Late-Maturing Citrus

Calcium, an essential plant nutrient and intracellular mineral element, plays a crucial role in fruit texture formation by participating in osmoregulation, influencing cell metabolism and division [17,36], while also reducing cell wall-degrading enzyme activity to maintain cell wall structure and function [45]. This study found that the calcium content in both the oil gland layer and the spongy layer of QJ, MRJ, and CJ initially increased and then decreased as peelability improved, with the correlation analysis revealing significant positive relationships between the calcium content and peel hardness, peel adhesion, and the CL and HCL contents, while showing significant negative correlations with TS and TA. At full maturity, CJ exhibited a significantly lower calcium content in both tissue layers compared to QJ and MRJ, indicating that reduced calcium levels influence the accumulation of cellular organic matter and cell wall polysaccharides, thereby affecting the cellular membrane and wall homeostasis and modifying the cell size and arrangement patterns, ultimately leading to decreased peel hardness and adhesion and facilitating peelability development, with the observed varietal differences in the calcium content accounting for peelability variations among the cultivars, consistent with calcium’s established role as a signaling molecule that crosslinks homogalacturonan to enhance pectin stability, inhibit polygalacturonase activity, and delay cell wall degradation [46].

### 3.5. Influence of Phytohormones on Peelability in Late-Maturing Citrus

Fruit texture modification is coordinately regulated by multiple phytohormones. Previous studies have demonstrated that ETH and ABA can modulate fruit firmness in strawberries [47] and blueberries [48] by regulating pectin and other cell wall polysaccharides, while phytohormone levels may also influence cell wall stability through the modulation of cell wall-degrading enzyme activities [49,50]. Our results showed that at full maturity, QJ exhibited significantly higher ABA and JA contents in both the oil gland layer and the spongy layer compared to MRJ (moderately difficult to peel) and CJ. Conversely, CJ displayed a significantly higher ETH content in its oil glands and a significantly higher SA content in its spongy layer than both QJ and MRJ. These hormonal differences were accompanied by the elevated expression of key biosynthesis genes (*GA30X8*, *ACS9*, *NCED1*, *NCED2,* and *NCED3*), consistent with previous findings [51,52]. These results indicate that the differential accumulation of ABA, ETH, SA, and JA at full maturity likely affects the final determination of peel hardness and adhesion, thereby contributing to the observed peelability differences among the QJ, MRJ, and CJ cultivars.

## 4. Materials and Methods

### 4.1. Test Site and Materials

The experiment was carried out in the Institute of Fruit Trees, College of Horticulture, Sichuan Agricultural University (30.88°’ N, 104.25°’ E) from June 2023 to October 2024. The fruit samples of QJ, MRJ, and CJ were collected from 8-year-old adult fruit trees on orange rootstocks in the same citrus orchard in Sunjia Village, Xie’an Town, Renshou County, Sichuan Province. The area has a subtropical monsoon humid climate with an altitude of 450–880 m and an average annual temperature of 17.4 °C. The average annual rainfall is 945.9 mm, and the average sunshine duration is 1196.6 h. The orchard employed sprinkler irrigation systems, typically operating from 9:00 PM to 6:00 AM, with the irrigation frequency being adjusted according to the prevailing weather conditions. Sampling began at 90 days after full bloom, and then 36 fruits of 7 developmental stages were selected every 30 days. The peels were stored at −80 °C for the experimental analysis.

### 4.2. Determination of Mechanical Indexes of Peel

Peel hardness was measured on the equatorial surface of the citrus fruit using a texture analyzer (Food Technology Corporation, Dulles Town Center, VA, USA) and a cylindrical probe TA/5 (diameter 5 mm). The texture condition setting conditions were as follows: the initial force was 0.75 N, the puncture speed was 1 mm/s, the return speed was 1 mm/s, the puncture distance was 25 mm, and the maximum resistance recorded by the texture analyzer was the peel hardness. Peel viscosity was measured using a texture analyzer (Food Technology Corporation, Virginia, USA) and a clamp probe. Each fruit sample was cut into blocks with an average growth of about 40 mm and a width of about 20 mm on the equatorial plane. The short side of the peel was fixed by the clamp, and the pulp on one side was pulled down vertically with tweezers until the peel and pulp were completely separated. The texture analyzer began to count when the tweezers clamped the pulp. The maximum resistance recorded by the texture analyzer was the peel adhesion.

### 4.3. Fruit Quality Determination

The fruit longitudinal diameter, transverse diameter, peel thickness, and pulp diameter were measured by vernier caliper. The TA content was measured using a sugar–acid all-in-one machine (ATAGO, Tokyo, Japan). The contents of TS and ST were measured by anthrone ethyl acetate and concentrated sulfuric acid reagent. The sugar components in the tissue were extracted by ethanol solution, and the sugar components were measured. The acid components were determined by high performance liquid chromatography. The determination conditions of high performance liquid chromatography were as follows: column temperature 25 °C, injection volume 10 μL, mobile phase 4% methanol aqueous solution, and flow rate 0.8 mL/min.

### 4.4. Paraffin Section Preparation and Cell Microscopic Observation

Saffron solid green paraffin sections were made by Seville Biotechnology Co., Ltd. (Wuhan, Hubei). An Olympus BH-2 optical microscope (Olympus, Japan) was used to observe and photograph the appropriate field of view at 20 times magnification, and Image J was used to measure the perimeter and area of pericarp cells.

### 4.5. Determination of Cell Wall Polysaccharide Content and Related Degradation Enzyme Activity

The cell wall material (CWM) was extracted from 1 g of dried and ground peel samples with dimethyl sulfoxide and acetone reagents. Then, 0.05 g of the CWM was added to sodium acetate twice and Na_2_CO_3_ once. The contents of WSP, ISP, and CSP were determined by carbazole colorimetry. Then NaOH and concentrated sulfuric acid were added to the precipitate to determine the contents of CL and HCL by anthrone colorimetry. Cx, PG, PL, and PME activities were measured using commercial kits.

### 4.6. Determination of Mineral Element Content

First, 0.5 g of dried and grinded peel samples were digested with nitric acid on an adjustable electric heating plate, and the contents of P, K, S, P, Zn, Ca, Mg, Fe, Cu, and Mn were determined by an atomic absorption spectrophotometer.

### 4.7. Determination of Plant Hormone Content

The contents of IAA, CTK, GA, ABA, and ETH were determined by high performance liquid chromatography (HPLC) from Sanshu Biotechnology Co., Ltd. (Nantong, China). Then, 100 mg of sample was accurately weighed, and 1.0 mL of pre-cooled 50% acetonitrile (ACN) aqueous solution was added. Subsequently, the sample was sonicated at 4 °C for 3 min, and then extracted at 4 °C for 30 min. After extraction, the sample was centrifuged at 12,000 rpm for 10 min at 4 °C, and the supernatant was collected for further processing and analysis. The results were based on high resolution mass spectrometry, and a qualitative analysis was performed by the identification of secondary mass spectrometry fragments. A quantitative analysis was performed by the external standard method.

### 4.8. Quantitative Reverse Transcription Polymerase Chain Reaction

RNA was extracted from peel samples using the chloroform–isopropanol method, cDNA was obtained using abm’s 5X All-In-One RT MasterMix reverse transcription kit, and a fluorescence quantitative polymerase chain reaction was performed using a Beijing Jumei Co., Ltd., commercial kit (Beijing Changping) and a fluorescence quantitative PCR instrument (Bio-Rad Laboratories, Inc, Hercules, CA, USA). Each reaction system used 10 μL and the reaction system included 5 µL 5 × M5 RT Super plus Mix, 3 µL ddH_2_O, 1 µL cDNA, and 1 µL 10 mM primer. Actin was used as an internal control. The 2^−ΔΔCT^ method was applied to calculate the relative gene expression.

### 4.9. Statistical Analysis

Statistical significance was evaluated using an analysis of variance (ANOVA) in SPSS 23.0 (IBM, Armonk, NY, USA), with a significance threshold set at *p* < 0.05. Origin 2021 (Origin Lab Corporation, Northampton, MA, USA) software was used to draw the figures.

## 5. Conclusions

The results of this study demonstrate that the peelability of QJ, MRJ, and CJ gradually decreases during fruit development and ripening, with all three cultivars exhibiting easier peeling characteristics at full maturity, following the order of QJ (most difficult to peel) > MRJ (moderate) > CJ (easiest). Throughout the phenological period, compared to MRJ and CJ, QJ showed several distinctive features: larger oil glands, more regularly shaped and uniformly sized spongy layer cells with a tighter arrangement, a lower accumulation of intracellular organic matter (including soluble sugars, titratable acids, and starch), and a higher content of cell wall polysaccharides (cellulose and hemicellulose) and calcium. These structural characteristics likely contribute to QJ’s superior peel integrity and most-difficult peelability. At full maturity, the observed differences in mineral elements and phytohormones may lead to cultivar-specific variations in organic matter accumulation and cell wall degradation, ultimately affecting their cellular morphology and arrangement and resulting in different peelability characteristics among these citrus cultivars. These results provide a reference for future studies on citrus peelability.

## Figures and Tables

**Table 1:** Changes in the external quality of three late-maturing citrus cultivars at different growth and development stages.

Cultivar	Days After Full Bloom	Vertical Diameter	Horizontal Diameter	Fruit Shape Index	Pulp Diameter	Total Peel Thickness
	(d)	(mm)	(mm)		(mm)	(mm)
QJ	90	53.92 ± 0.98 a	53.66 ± 1.08 a	1.00 ± 0.00 a	40.93 ± 1.17 a	6.37 ± 0.07 a
	120	63.19 ± 0.65 a	68.59 ± 1.05 a	0.92 ± 0.02 a	57.06 ± 0.67 a	5.76 ± 0.31 a
	150	72.80 ± 0.45 a	85.54 ± 1.07 a	0.85 ± 0.01 a	73.44 ± 1.03 a	6.05 ± 0.05 a
	180	74.15 ± 1.00 a	83.80 ± 0.46 a	0.88 ± 0.01 a	71.11 ± 0.73 a	6.34 ± 0.29 a
	210	74.94 ± 1.46 a	85.06 ± 1.37 a	0.88 ± 0.01 a	72.13 ± 1.94 a	6.47 ± 0.36 a
	240	83.61 ± 0.94 a	95.15 ± 1.24 a	0.88 ± 0.01 a	82.18 ± 0.68 a	6.49 ± 0.37 a
	270	84.22 ± 0.30 a	96.93 ± 0.79 a	0.87 ± 0.01 a	84.18 ± 0.84 a	6.37 ± 0.06 a
MRJ	90	40.59 ± 1.50 b	45.44 ± 1.57 b	0.89 ± 0.01 b	39.87 ± 1.20 ab	2.79 ± 0.18 c
	120	47.89 ± 0.13 c	55.60 ± 0.30 b	0.86 ± 0.00 b	51.67 ± 0.25 b	1.96 ± 0.03 c
	150	51.53 ± 1.25 c	61.90 ± 2.05 c	0.83 ± 0.02 a	58.08 ± 1.82 c	1.91 ± 0.13 c
	180	55.61 ± 0.48 c	64.36 ± 0.95 c	0.86 ± 0.02 a	60.31 ± 0.85 b	2.03 ± 0.06 c
	210	58.93 ± 1.28 c	66.23 ± 1.00 c	0.89 ± 0.01 a	61.57 ± 0.93 b	2.33 ± 0.03 c
	240	56.73 ± 2.41 c	64.93 ± 1.91 c	0.87 ± 0.01 a	59.75 ± 2.17 c	2.59 ± 0.16 c
	270	58.89 ± 0.60 c	66.88 ± 0.20 c	0.88 ± 0.01 a	61.07 ± 0.14 c	2.90 ± 0.06 c
CJ	90	41.95 ± 0.78 b	45.98 ± 0.70 b	0.91 ± 0.00 b	37.86 ± 0.97 b	4.06 ± 0.14 b
	120	51.32 ± 1.33 b	56.92 ± 1.09 b	0.90 ± 0.01 a	49.87 ± 1.04 c	3.53 ± 0.08 b
	150	55.23 ± 0.88 b	68.29 ± 0.43 b	0.81 ± 0.01 b	62.35 ± 0.41 b	2.97 ± 0.13 b
	180	62.33 ± 1.67 b	78.03 ± 0.97 b	0.80 ± 0.01 b	71.21 ± 1.02 a	3.41 ± 0.17 b
	210	62.78 ± 0.54 b	77.78 ± 1.05 b	0.81 ± 0.01 b	70.02 ± 1.32 a	3.88 ± 0.25 b
	240	64.60 ± 1.87 b	80.37 ± 1.27 b	0.80 ± 0.01 b	72.51 ± 0.98 b	3.93 ± 0.20 b
	270	64.46 ± 1.11 b	78.42 ± 0.70 b	0.82 ± 0.02 b	70.50 ± 0.63 b	3.96 ± 0.04 b

Note: Data are presented as mean ± standard deviation (n = 3). Different lowercase letters following similar data of different varieties in the same period indicate significant differences according to Duncan’s test (*p* < 0.05).

## Data Availability

Data are contained within the article.
